# Nerve Training

**Published:** 1898-06

**Authors:** 


### Nerve Training.

The question of the susceptibility of the nervous system for training has recently been discussed, and a great deal of difference of opinion appears to exist as to the possibility of training the nerves. According to the most comprehensive opinion, a great deal depends on the owner of the nerves. It is possible to train certain classes and conditions, while others are hopelessly unsusceptible. The will of the individual, the pliability, or rather the impressionability, has everything to do with successful nerve training. As a matter of fact, the desire to be trained must be present first of all. It comes from within, and prompted by the desire of the individual, a course of training may bring about the happiest results. Training nerves against the will of the patient reminds one of the old adage of convincing a man against his will—"He is of the same opinion still."—*Indian Lancet.*

